# The institutional foundations of surf break governance in Atlantic Europe

**DOI:** 10.1007/s11127-021-00929-3

**Published:** 2021-09-15

**Authors:** Martin Rode

**Affiliations:** Departamento de Economía, Universidad de Navarra, Campus Universitario, 31009 Pamplona, Navarra Spain

**Keywords:** Social trust, Cultural tightness-loosness, Property rights, Common pool resources, D71, K11, Q26, Z13

## Abstract

The sport of surfing is best enjoyed with one rider on one wave, but crowding makes that optimal assignment increasingly hard to attain. This study examines the phenomenon of surf localism, whereby competitors are excluded from waves by intimidation and the threat of violence. An alternative way to accommodate crowds is contained in the surfer’s code, which sets informal rules and self-enforced regulations to avoid conflict in the water. Both regimes establish property rights over common pool resources with no state intervention, creating a setting wherein users face the question of cooperation or conflict. The disposition to cooperate and follow norms has been shown to vary substantially across different cultures, though. Employing data from over seven hundred surf spots on the European Atlantic coast, this study reports evidence that certain informal cultural norms significantly reduce the probability of violent exclusion, while formal state institutions mostly are irrelevant. The results also indicate that informal norms become more important with greater resource quality and, possibly, with increasing scarcity.

## Introduction

The problem of of open access resources in the absence of well-defined property rights can more or less be considered a classic topic in public economics. Ensuing debates have often concentrated on the question of how much government intervention is necessary to guarantee sustainable exploitation, when formal property rights are not well defined, and cannot easily be created or monitored (Pigou 2013). As Demsetz (1974) argues, property rights will be created endogenously by the actual users when the benefits of developing and enforcing them exceed the costs, with or without the participation of government. Examples of extralegal governance are numerous and well documented in the relevant literature for a very diverse array of human activities (Radford 1945; Acheson 1988; Ostrom 1990; Benson 2006; Leeson 2007; Powell and Stringham 2009; Stewart 2009; Skarbek 2014).

Following studies by Nazer (2004), Kaffine (2009), and Mixon (2014), this paper argues that the sport of surfing is an excellent application for studying open access resources in the context of formally absent property rights and little to no state intervention. Given the way that waves are ridden by surfers, the sport is best enjoyed with one rider on one wave. As rideable waves are in limited supply, though, it essentially converts surf breaks into a common pool resource problem that breeds substantial potential for (violent) conflict. Recently, the continuous growth of surfing has crowded many breaking waves, increasing the possibility for interactions of individuals in the water, but also making them much more anonymous and prone to rivalry (de Alessi 2009; Ponting and O’Brien 2015).

One way to deal with the common pool problem of wave assignment, is the quite well-known phenomenon of *surf localism*. Essentially, localism is the territorial behavior of resident (or habitual) surfers over surf breaks (Usher and Gómez 2016), oftentimes through intimidation, the threat of violence, or sometimes the actual use of force against persons or property. For the purpose of this paper, territoriality excludes potential competitors from a common pool resource, wherein only group members are permitted access to entire breaks, or certain sections of it. Numerous examples of such exclusionary surf localism exist across the world, for example the infamous Lunada Bay in Palos Verdes Estates of Southern California^1^, Tamarin Bay on the island of Mauritius, or Rodiles in Northern Spain. The idea of shared wave ownership by certain groups might seem far fetched to non-surfers, but it is an ongoing source of friction in the sport, which also is subject to constant evolution. For instance, during the recent Covid-19 crisis, local surfers enforced municipal movement restrictions often, because they served their interests in reducing crowd pressure at local breaks.^2^

An alternative way of accommodating crowds in the water is the *surfer’s code*, which sets informal rules and self-enforced regulations to avoid conflict in the water. The basics of the code are known by all regular surfers (Nazer 2004), consisting of a simple set of rules that assign wave priority, paddling paths to avoid collision, and condemn drop-ins when waves already have been rightfully claimed.^3^ Taking turns and respecting local surf communities likewise are important extended aspects of the code, whereby local surfers automatically are favored by greater knowledge of wave conditions. If the rules are respected, the bottleneck problem of wave assignment can be managed quite efficiently, even when crowds are substantial (Nazer 2004).

Ideally, organized local surfers can act as enforcers of universal surfing etiquette and that happens sometimes (Beaumont and Brown 2016). Unlike the exclusionary localism descibed above, the arrangement does permit visiting surfers to access other breaks, as long as rules are followed. Still, once local surfers overcome the barriers to group formation (Mixon 2018), they remain prone to falling prey to the logic of any state-like institution, namely effectively organizing violence creates incentives to practice predatory behavior towards non-organized individuals or groups (Skarbek 2016; Benson 2020). Following Smith (2002), the question of exercising strong localism or the surfer’s code to manage crowds thus is, one of exclusion versus collective rule-following.

According to Boettke et al. (2015), spontaneously emerging institutions that exclude or govern ultimately reflect the preferences of local actors. Those preferences vary quite substantially across different cultures, though (Hofstede 2011). In that context, especially social trust (Putnam et al. 1993; Sønderskov 2011) and the tightness of cultural norms (Gelfand et al. 2006, 2011) have been identified as central elements that determine compliance or defection from informal rules. Alternatively, formalized and well-working legal systems theoretically could take on that role, potentially even crowding-in civic behavior over the long-run (Berggren and Jordahl 2006; Herreros and Criado 2008). As such, the functioning of institutions, either formal or informal, therefore should influence substantially how surf spots are governed by their users, determining whether exclusion actually is practiced by locals or not.

A series of related studies by Kaffine (2009); Mixon (2014); Mixon and Caudill (2018); Mixon and Sankaran (2019) already have inquired empirically into the determinants of surf localism. Still, in neither case has the role of institutions that facilitate cooperation and conformity with norms, either formal or informal, been incorporated to date. The present paper does so, accounting explicitly for the fact that different property rights’ regimes reflect the relative costs and benefits of their creation (Demsetz 1974), but also the cultural preferences of those who enforce them in one way or another (Ostrom 1990).

In doing so, the paper contributes to several strains of literature: First, it is related to recent studies of the relationship of customary law and state enforced law. Following Gutmann and Voigt (2020), given the importance of informal law in many societies, it is striking how little scholars actually know about its compatibility with the rule of law. The governance of surf breaks can be considered as a very good example of that relationship. Second, Powell and Stringham (2009) highlight how governments in many developing countries often are missing in action when it comes to protecting property rights. Still, many societies have found workable solutions to the ensuing problems; such decentralized, spontaneously emerging orders frequently may be more viable than externally funded state-building projects (Powell and Stringham 2009; Börzel and Risse 2016). The present paper studies how social order is established and how formal and informal rules contribute thereto. Third, the increase in surf tourism to many developing countries often sparks local conflicts over surf breaks that carry substantial potential for resort to violence (Nazer 2004; Ponting and O’Brien 2015; Usher and Gómez 2016). Uncovering the institutional foundations that undermine the viability of governing surf breaks in an efficient manner therefore also may make a substantive contribution to the successful resolution of such conflicts, facilitating sustainable exploitation of local breaks as a resource for surf tourism.

By collecting and analyzing data from 750 surfing venues scattered over 10 different countries and some 29 regions on the European Atlantic coast, this study reports evidence that regional social trust reduces the probability of violent exclusion in an open access setting with scarce resources, while formal legal systems largely are irrelevant. The findings indicate further that the tightness-looseness axis of cultural norms interacts with resource quality to a substantial degree, while the relevance of social trust in preventing exclusionary localism increases with more resource scarcity.

The remainder of the paper is organized as follows: Sect. 2 reviews briefly the relevant literature on culture, institutions, and property rights, as well as related studies on surf localism. Section 3 describes the data and the different control variables. Section 4 describes the estimation procedures employed and comments on the empirical results, while Sect. 5 concludes.

## Related literature

As Coase (1960) highlights, social costs may stand in the way of compliance with formal and informal rules, despite shared interest in reaching mutually beneficial agreements on common-pool resource use. In that context, it nowadays is well-established that governance outcomes depend heavily on culture (Hofstede 2011), with particularly importance assigned to mutual cooperation and norm compliance.

Here, two concepts stand out in the empirical literature: First, social capital and social trust are central elements that determine horizontal cooperation between individuals (Putnam et al. 1993; Sønderskov 2011). It is important to mention that several empirical studies by Uslaner (2002); Bjørnskov and Sønderskov (2013), and Bjørnskov (2019) have attributed the positive cooperation effects of social capital largely to the underlying factor of social trust, which also is on what the present paper will focus. In that sense, social trust has been shown to be an important element in explaining a wide variety of desirable social outcomes that hinge on participation, such as the performances of formal institutions (Boix and Posner 1998), economic growth (Knack and Keefer 1997; Zak and Knack 2001; Horváth 2013)), total factor productivity (Bjørnskov and Méon 2015), large scale economic reforms (Berggren and Bjørnskov 2017), or welfare state configuration and economic inequality (Bjørnskov and Svendsen 2013; Bergh and Bjørnskov 2014).

A willingness to cooperate, as expressed in a high level of social trust, therefore also should affect the likelihood of observing exclusionary localism at surf breaks. Potentially, the connection can happen in several ways: On the one hand, a high level of social trust could facilitate the generalized use of the surfer’s code over exclusion and intimidation, thereby potentially reducing the probability of localism (Reeskens and Wright 2013; Pitlik and Rode 2020, cf.).^4^ On the other hand, it might induces natives surf gangs to practice effective forms of fencing out visitors more viable, increasing the likelihood of that response (Numerato and Baglioni 2012; Uribe 2014, cf.). The second option nevertheless is much less likely in the setting at hand, as it rather exhibits strong features of high in-group trust, but low generalized trust between groups (Banfield 1967; Putnam 2001).

Alternatively, formalized and well-working legal systems theoretically can take on the role of facilitating mutual cooperation. Nazer (2004) also highlights that state intervention, in theory, possibly combats the violent outcomes of exclusionary localism. Still, it is an open question how many serious injuries from fights over waves actually will result in criminal charges being filed.^5^ An associated discussion concerns the question, whether well-working legal systems crowd-out (Lowes et al. 2017) or crowd-in (Herreros and Criado 2008) cooperative civic behavior. Broadly following the latter logic, Berggren and Jordahl (2006); Cassar et al. (2014); Martinangeli et al. (2020) find that legal quality predicts social trust. As such, well-working legal systems may not really be regarded as potential alternative dispute-resolution mechanisms for distributional conflicts over the common pool resource of surf waves, but they possibly could contribute to stronger dispositions toward mutual cooperation in the long run.

Second, the concept of norm tightness versus looseness tries to explain the relative importance that different cultures place on compliance with rules. Following Gelfand et al. (2006), tightness-looseness consists of the strengths of social norms and sanctioning, including tolerance for norm deviance. So, while “tight” cultures exhibit strong norms and low tolerances for deviant behavior, “loose” cultures have rather weak norms and high tolerances for deviant behavior. As such, the cultural tightness-looseness concept bears some relation to the individualism-collectivism approach of Hofstede (2011), although tightness-looseness really isn’t concerned with the content of any specific cultural rules themselves, but only with the degree to which people believe that rules, once established, should be respected.

A priori, one might expect tight cultures to rely more heavily on decentralized norms, such as the surfer’s code. Still, research has shown that tight cultures are more likely to be politically autocratic, characterized by intensive reliance on policing of society to guarantee norm compliance, even under democratic governance systems (Gelfand et al. 2011). That tendency probably also would make the appearance of exclusionary localism more likely in tight cultures because individuals in such societies place comparatively more emphasis on the establishment of order to resolve situations of distributional conflict. Following Acheson (1988), Nazer (2004) believes that the extent of close-knittedness (i.e., tightness) among locals is a determinant of how vigorously they defend “their” territories. In that sense, tight cultures also seem to be better able to respond to rapidly changing external environments by cooperating much more quickly under threat, as exhibited by the much lower mortality rates of tight societies during the late Covid 19 pandemic (Gelfand et al. 2021).

Regarding the empirical correlates of localism, a series of related studies by Kaffine (2009); Mixon (2014); Mixon and Caudill (2018); Mixon and Sankaran (2019) already have inquired empirically into its determinants, mainly centering on the roles of crowd pressure, resource quality, and exogenous threats (i.e., sharks). For the most part, those studies focus exclusively on the US state of California, though, while Mixon and Caudill (2018) rely on only a few cross-country observations. Generally, all of the aforementioned studies rely on relatively small datasets and the role of institutions, either formal or informal, have not been incorporated into any of them. Employing dummy variables, Mixon (2014) and Mixon and Sankaran (2019) find significant differences for a variety of regions in California, which potentially can be interpreted as outcomes of variations in cultural traits and institutions. Likewise, Mixon and Caudill (2018) rely on continental-level dummies, finding significant differences across big wave locations. To some extent, then, these studies anticipate the results presented in the following, although without any clear conception of how formal and informal institutions should be captured, or how they might affect surf break governance.

Finally, another strand of theoretical literature has examined the conditions under which locals organize effectively to fence out competitors from surf breaks (Rider 1998; Mixon 2018). That literature is based on game-theoretical considerations and bears a clear relationship to conceptual studies on anarchy and nascent state formation (Buchanan 1975; Olson 1993; Holcombe 2017, cf.).^6^ The bottom line that can be extracted from those previous studies is that especially heavy congestion will create strong incentives to exclude competitors from the common pool resource of waves, analogous to the incentives that spurn the development of private property rights under situations of anarchy (Kurrild-Klitgaard and Svendsen 2003; Benson 2006; Skarbek 2014; Mixon 2018, e.g.). In that sense, it could even be argued that a successfully localized surf venue presents important features of club goods (Buchanan 1965), wherein the marginal benefit from one additional member of a group of locals is equal to the marginal congestion costs imposed on others by that same member.

## Data and variables

Information on all surf venues observed herein was obtained from the participatory open-access website www.wannasurf.com. That site provides detailed travel reports for thousands of surf spots around globe, with most of the information coming from local users. Reports are confirmed further by designated area representatives in order to avoid possible bias. Nevertheless, local users (and area representatives) might have incentives to deter crowds by manipulating figures. As such, the information contained in the resulting dataset was double checked using the forecasting and travel guide website www.magicseaweed.com, as well as the print version of a well-known and long standing surf guide by Fitzjones (1998), of which updated versions also are available. The latter two sources are commercial products for which manipulation incentives obviously are much less problematic. As a consequence, several surf spots are not counted as localized, despite being designated as such in the main source.^7^ Practically all available surfing venues in Europe that face the open Atlantic Ocean were coded for the purpose of the present paper.

Similar to the studies by Kaffine (2009), Mixon (2014), Mixon and Sankaran (2019), the main outcome variable herein is a measure of surf localism for individual wave breaks. As highlighted by the studies’ authors and in the discussion above, localism can take several different forms: On the most extreme end, it can range from a rule enforcing access to non-locals, mainly by relying on non-verbal communication, at times coupled with explicit warnings, all the way to verbal abuse and threats (and sometimes violence) to make non-local surfers leave a break. The papers mentioned above try to capture such differences by creating an index of localism that ranges from 1 to 5, where higher values indicate stronger localism.^8^ Corresponding information unfortunately is not systematically available for a large variety of surf spots along the European Atlantic coast. Following Bennett (2004) and Nazer (2004), it also is somewhat unclear whether rule enforcing and exclusionary localism really should be measured on the same scale, as both differ fundamentally in their approaches to non-local surfers.

To capture *localism* in this paper, I code a dummy from the information available in the aforementioned sources. The binary variable takes a value of one for all surf spots that are very localized, in the sense that localism is exclusionary for entire breaks or well-defined sections of breaks, and zero otherwise. That variable is supposed to capture only the type of localism that is expressed in the popular phrases: “If you don’t live here, don’t surf here” or “Locals only”. As such, the rule enforcing version of localism, explicitly permitting outsiders to surf under the governance of surfing etiquette, is not captured in this paper. For most surf spots, the type of localism present at the respective locations is very obvious from the comments across all three sources. If in doubt, the surfing venue was counted as a non-localized spot.

Further following the work of Kaffine (2009), Mixon (2014), Mixon and Caudill (2018), Mixon and Sankaran (2019), a number of controls for each surfing spot are coded from the same sources: *Wave quality* attempts to capture the character of the resource available. It is based on a ten-point user rating, which is converted into an ordinal 1–3 scale for the present purpose; higher values indicate comparatively better waves. Likewise, *congestion* reflects crowd pressures during the week and on weekends, which I measure on an ordinal scale from 1 to 3, where higher values indicate more congestion. The level of surfing skills required at each venue, is captured by a binary variable *ability*. Originally based on a four-point scale, it is converted to a dummy for the present purpose, so as to reduce the obvious correlation with wave quality. A one indicates that comparatively more ability is required to surf the break, and zero otherwise.

Overall, previous papers find better wave quality, more congestion, and higher levels of surfing ability to be associated significantly with the prevalence of localism. While congestion and ability potentially are endogenous to localism, both Kaffine (2009) and Mixon (2014) highlight that in surfing, resource quality (i.e., waves) must be exogenous. Given the frequently strong association between resource quality and property rights protections, the causal relation between both frequently is unclear in other studies (Acheson 1988, cf.).

*Access* measures the ease of being able to surf a spot on an ordinal scale from 1 to 4, reflecting whether the spot has direct access, if it is easy to find, and whether it offers public access. Higher values indicate easier access to the corresponding break. Relatedly, *urban* is a dummy set equal to one, if a break is an urban area, and zero otherwise. *Tourism* and *pollution* are two additional binary variables that are set equal to one, if comparatively high levels of tourism/pollution are present at the break, zero otherwise.

Generally speaking, one would expect venues with easier access, located in urban areas, and with comparatively more tourism also to be more prone to develop exclusionary localism. In turn, the association with pollution can be expected to be negative, as surfing polluted spots on a regular basis potentially can present serious health risks. Previous findings by Kaffine (2009) and Mixon (2014) did not produce any consistent results with respect to access and pollution, though.

Additionally, two alternative variables to capture wave quality are coded: *Frequency* captures the regularity with which a break can be surfed, measured on an ordinal scale from 1 to 3. Higher values indicate more regularly breaking waves, roughly corresponding to a low-, middle-, and high frequency. The variable *length* attempts to reflect the duration of a ride on the wave, from the first breaking point to its end close to shore, as longer rides generally are preferred by all surfers. It also is measured on an ordinal scale from 1 to 3, for which higher values indicate longer waves.^9^

In order to capture regional level culture, or informal institutions, two variables are constructed from data in the European Social Survey (ESS). This survey has been conducted across European countries on a bi-annual base since 2001, attempting to capture attitudes, beliefs, and behavioral patterns of the general population in face-to-face interviews.

First, *social trust* is captured as regional averages of the following ESS survey question: “Most people can be trusted, or you can’t be too careful?” Respondents answer on a 1–10 scale; increasing values indicate more social trust. According to Bjørnskov (2019), the history of relying on responses to that question in social science research is long and a broad consensus has emerged that it captures meaningful differences in beliefs at both the individual and societal levels. For example, it has been asked in various US surveys since the late 1950s and in all waves of the World Values Survey beginning in 1981.

When asking the question, it nonetheless is not made clear to respondents whom to trust, in which situations, or under which circumstances (Bjørnskov 2019). Potentially, that ambiguity could mean that respondents don’t distinguish between generalized trust or more particularized forms of trust at the moment of answering. However, several different tests suggest that the trust question captures individuals’ beliefs that strangers can be trusted quite well: For instance, in-depth interviews reported in Uslaner (2002, 2007) show that the simple question picks up trust in random people, about whom the respondent has no specific information. Furthermore, Knack and Keefer (1997) and Bjørnskov (2021) show that trust scores at the national level correlate strongly with return rates in a wallet-drop experiment, which cannot simply reflect the quality of formal enforcement institutions.

Second, I follow Gelfand et al. (2011) to capture *tight-loose* cultures by calculating regional averages of four different ESS survey questions, which are designed to measure the relative importance of certain values: (1) “Important to do what is told and follow rules”, (2) “Important to behave properly”, (3) “Important to follow traditions and customs”, and (4) “Important to make own decisions and be free” (inverted). The responses to the original questions all are measured on a ten-point scale. In order to make the index more comparable with Gelfand et al. (2011), I convert the averaged measure into an index between 0 and 1, where higher values correspond to tighter cultural norms.

Finally, I rely on the European Quality of Government index by Charron et al. (2019) to measure *legal quality* at the regional level. That variable attempts to capture the quality and impartiality of formal institutions in European regions, as well as the perceived level of institutional corruption. It is measured on a scale from 0 to 100, where higher values indicate comparatively better functioning formal institutions.

Given that the surfing venues data roughly reflects conditions between 2010 and 2020, and that social trust (Bergh and Öhrvall 2018; Bjørnskov et al. 2021) and formal judicial institutions (Sobel and Coyne 2011) both generally are slow to evolve, the macro-variables are calculated from regional averages of all nationally available ESS waves between rounds 4 and 8. Those survey waves correspond to the years between 2008-2009 and 2016-2017, while legal quality is measured for the year 2017. The resulting multilevel cross-sectional dataset contains information for 750 individual surf spots in 10 countries^10^, and regional level data on social trust and legal quality for some 29 regions. Table 1 provides summary statistics for all variables entered in the following estimations.

**Table 1:** Summary statistics

Variables	Mean	SD	Min	Max	Obs
Localism	0.212	0.409	0	1	750
Congestion	1.524	0.663	1	3	750
Ability	0.329	0.470	0	1	750
Access	1.304	0.569	1	4	750
Tourism	0.355	0.479	0	1	750
Urban	0.317	0.466	0	1	750
Pollution	0.179	0.383	0	1	750
Wave quality	1.872	0.611	1	3	750
Frequency	1.831	0.667	1	3	750
Length	1.457	0.576	1	3	750
Social trust (Reg.)	4.977	0.774	3.443	6.907	29
Tight-loose (Reg.)	0.494	0.035	0.421	0.566	29
Legal quality (Reg.)	63.571	10.580	39.940	79.950	29

## Estimations and findings

In order to test whether social trust and legal quality affect the prevalence of exclusionary surfing localism formally, I estimate the following non-linear (probit) models:

1 $$\begin{aligned} (Localism_{ij}&= 1 | X_{ij}, Trust_{j}, Tightloose_{j})\nonumber \\&= Phi(\beta _1 X_{ij} + \beta _2 Trust_{j}+ \beta _3 Tightloose_{j}) \end{aligned}$$

2 $$\begin{aligned} (Localism_{ij}&= 1 | X_{ij}, Trust_{j}, Tightloose_{j}, Legal_{j}) \nonumber \\&= Phi(\beta _1 X_{ij} + \beta _2 Trust_{j} + \beta _3 Tightloose_{j} + Legal_{j}) \end{aligned}$$

where $Localism_{ij}=1$ expresses the probability that surf spot *i* in region *j* is characterized by exclusionary localism. *Phi* is the standard normal cumulative distribution, while a set of covariates $X_{ij}$ controls for other factors that might drive the probability of observing a localized surf spot. Variables $Trust_j$, $Tightloose_j$, and $Legal_j$ specify the regional level indicators of formal and informal institutions. Standard errors are clustered at the regional level in all estimations.

Because the institutional measures reflect regional averages, one also can be pretty confident on the direction of causality: On the one hand, we can expect the behavior of individuals frequenting surf breaks to be driven substantially by local culture. At the same time, all of those individuals are subject to the same formal institutions. On the other hand, the probability that the presence of localism at certain surf spots would influence culture among the general population of a region significantly can be considered as rather unlikely. That is even more so for the quality of formal legal systems and executive enforcement. Of course, one cannot exclude the possibility that social trust, tightness-looseness, and formal legal institutions are themselves reflections of other underlying (cultural) factors that are not captured in the model and likewise drive individual dispositions towards localism. Still, it is unclear at present what those factors should be; they certainly don’t make the direct influence of localism on any of the macro-variables more likely. In that sense, a robustness-check with regional fixed effects also yields very similar results.^11^ The estimations therefore should approximately reflect the causal effect of formal and informal regional institutions on localism.

The findings from the baseline regressions are displayed in Table 2, where the full set of controls are displayed for a variety of different models. Columns (1–5) correspond to equation 1, entering only the cultural variables as primary controls. In turn, columns (6–10) correspond to equation 2, where estimations are repeated with legal quality introduced to all of them. It should be noted that social trust and legal quality correlate at 0.8 in the data, while the cultural tight-loose measure practically is unrelated to both. All three institutional variables are introduced as continuous to the regressions. In contrast, all controls are broken down into individual dummy variables to facilitate interpretation; the lowest value always is the omitted category.

Commenting first on the controls, column (1) employs wave quality only, finding both dummies to be statistically significant at conventional levels and to enter the equation with the expected positive signs. Entering additional controls in columns (2) and (3) shows that easier access is only partially significant for explaining localism, while wave quality loses statistical significance at the introduction of ability in column (3), which in turn is statistically significant and positive. Given the similarity of both concepts, that result does not really come as a big surprise. Interestingly, we also find pollution to be insignificant in explaining the probability of observing exclusionary localism in column (3). Column (4) substitutes wave quality for wave frequency and length, which nonetheless both are insignificant as well. Finally, the model in column (5) substitutes access for urban and congestion for tourism; we again find only the latter to be positive and statistically significant, thereby confirming results from columns (2–4). It is notable that all findings likewise very much align with the results of Kaffine (2009), Mixon (2014), Mixon and Sankaran (2019) for California, providing additional indications of their validity.

In columns (1–5) of Table 2, the impact of social trust is negative and significant, meaning that comparatively more regional social trust reduces the probability of observing exclusionary localism, while the tight-loose measure always is insignificant. Following the interpretation of Nazer (2004), all else equal, social trust seems to facilitate the generalized use of the surfer’s code over intimidation and exclusion in resolving the common pool resource conflict over surfing waves.

Introducing legal quality in columns (6–10) of Table 2 produces some further noteworthy results: All basic control variables behave in an analogous manner, highlighting the significantly positive effect of better wave quality, more congestion (or tourism), and greater surfing ability on the probability of observing exclusionary forms of localism at surf spots across Atlantic Europe. In turn, regional legal quality never is statistically significant. Also, social trust now is statistically significant in only two of the five models, namely (1) and (2), albeit always carrying the expected negative sign. Interestingly, legal quality becomes statistically significant and returns the expected negative sign in all models, once social trust is excluded from the estimations.^12^ Essentially, that finding merely reflects the relatively high correlation of both variables. Still, the results indicate tentatively that the willingness to cooperate essentially is more important for the dearth of localism than the quality of formal enforcement institutions, despite the fact that well-working legal systems may facilitate the development of social trust in the long-run (Berggren and Jordahl 2006, cf.).

**Table 2:** Determinants of localism

Variables	(1)	(2)	(3)	(4)	(5)	(6)	(7)	(8)	(9)	(10)
	Culture	Culture	Culture	Culture	Culture	Cult.+Leg.	Cult.+Leg.	Cult.+Leg.	Cult.+Leg.	Cult.+Leg.
Social trust	− 0.332***	− 0.300***	− 0.277***	− 0.266***	− 0.292***	− 0.225*	− 0.254**	− 0.175	− 0.180	− 0.145
	(− 3.07)	(− 3.17)	(− 2.86)	(− 2.69)	(− 2.77)	(− 1.83)	(− 2.01)	(− 1.07)	(− 1.07)	(− 0.95)
Tight-loose	− 1.535	− 1.217	− 1.081	− 1.031	− 1.920	− 0.475	− 0.745	− 0.0446	− 0.163	− 0.440
	(− 1.18)	(− 0.96)	(− 0.69)	(− 0.64)	(− 1.30)	(− 0.22)	(− 0.35)	(− 0.02)	(− 0.07)	(− 0.19)
Legal quality						− 0.00848	− 0.00371	− 0.00805	− 0.00681	− 0.0116
						(− 0.80)	(− 0.38)	(− 0.70)	(− 0.59)	(− 1.13)
Congestion2		0.623***	0.678***	0.689***			0.621***	0.674***	0.687***	
		(4.66)	(4.75)	(4.97)			(4.72)	(4.79)	(5.02)	
Congestion3		1.409***	1.425***	1.437***			1.405***	1.415***	1.428***	
		(8.87)	(8.97)	(9.24)			(8.96)	(9.22)	(9.59)	
Access2		0.138	0.00732	0.00741			0.136	0.00237	0.00276	
		(1.04)	(0.06)	(0.06)			(1.01)	(0.02)	(0.02)	
Access3		0.665**	0.513**	0.511**			0.664**	0.507*	0.504**	
		(2.47)	(1.98)	(2.08)			(2.44)	(1.93)	(2.03)	
Access4		− 0.265	− 0.446	− 0.372			− 0.250	− 0.415	− 0.351	
		(− 0.49)	(− 0.89)	(− 0.73)			(− 0.47)	(− 0.85)	(− 0.70)	
Ability1			0.508***	0.544***	0.521***			0.515***	0.549***	0.532***
			(4.99)	(5.47)	(4.99)			(4.90)	(5.41)	(5.07)
Pollution1			0.0757	0.0783	0.125			0.0902	0.0908	0.142
			(0.40)	(0.43)	(0.58)			(0.47)	(0.48)	(0.66)
Frequency2				0.0989					0.0972	
				(0.98)					(0.95)	
Frequency3				0.0340					0.0315	
				(0.21)					(0.19)	
Length2				− 0.0433					− 0.0431	
				(− 0.30)					(− 0.29)	
Length3				− 0.0943					− 0.0865	
				(− 0.33)					(− 0.30)	
Wave quality2	0.271*	0.241*	0.170		0.200	0.280*	0.245*	0.178		0.214
	(1.90)	(1.84)	(1.22)		(1.37)	(1.94)	(1.85)	(1.25)		(1.43)
Wave quality3	0.413**	0.334**	0.157		0.147	0.415**	0.334**	0.155		0.148
	(2.20)	(2.12)	(0.91)		(0.75)	(2.13)	(2.09)	(0.87)		(0.72)
Tourism1					0.720***					0.711***
					(6.06)					(6.30)
Urban1					0.0160					0.0305
					(0.16)					(0.30)
Observations	750	750	750	750	750	750	750	750	750	750
Prob	0.0113	0	0	0	0	0.0260	0	0	0	0
$Chi^2$	13.00	198.4	168.6	171.5	149.0	12.73	235.5	224.6	248.9	141.1
$R^2$	0.0376	0.136	0.159	0.158	0.114	0.0386	0.136	0.159	0.158	0.116

Standard errors in parentheses: $${*}p<0.10$$, $${**}p<0.05$$, $${***}p<0.01$$

As is well-known, coefficients obtained from non-linear probit estimations reflect probabilities, making the magnitude of changes in independent variables on the outcome somewhat difficult to interpret. To that end, Table 3 shows marginal probabilities of observing exclusionary localism for all independent variables, corresponding to the results from Table 2.^13^ Similar to the magnitudes of effects found by Kaffine (2009); Mixon (2014) based on ordered probit estimations, Table 3 shows for a middle level of wave quality to increase the probability of observing strong localism by about 6 or 7 percentage points, while a high level of wave quality is associated with an 8–11 percentage point higher probability. Regarding congestion, Table 3 shows that at a middle level of congestion at surf breaks, the probability of strong localism increases by about 16–17 percentage points, while a high level of congestion is associated with a 43–45 percentage point larger probability of localism. Finally, more surfing ability makes localism more likely by about 13 or 15 percentage points, while tourism makes exclusion more probable by about 20 percentage points.

Regarding our primary variables of interest, a one standard deviation increase in social trust reduces the probability of observing exclusionary localism by somewhere between 6 and 9 percentage points. The underlying variation in social trust that causes the difference roughly corresponds to the real-world difference between Southern Norway and the Western Netherlands on the higher end, or Central Portugal and Northwestern Spain on the lower end. Correspondingly, making a hypothetical move from the lowest possible level of social trust in Atlantic Europe (the Algarve in Portugal) to the highest possible level (Western Denmark) would reduce the probability of observing exclusionary localism by some 40 percentage points. In absolute terms, the total impact of social trust is, therefore, comparable to that of congestion. So, when looking at the case of surfing, the purely numerical impact of informal institutions on the probability of observing exclusion in common pool resource conflicts is far from trivial.

**Table 3:** Determinants of localism (Marginal effects)

	(1)	(2)	(3)	(4)	(5)	(6)	(7)	(8)	(9)	(10)
Variables	Culture	Culture	Culture	Culture	Culture	Cult.+Leg.	Cult.+Leg.	Cult.+Leg.	Cult.+Leg.	Cult.+Leg.
Social trust	− 0.0925***	− 0.0751***	− 0.0673***	− 0.0647***	− 0.0748***	− 0.0628*	− 0.0635**	− 0.0426	− 0.0438	− 0.0370
	(− 3.51)	(− 3.41)	(− 3.01)	(− 2.82)	(− 2.94)	(− 1.81)	(− 1.98)	(− 1.06)	(− 1.06)	(− 0.95)
Tight-loose	− 0.428	− 0.304	− 0.263	− 0.251	− 0.491	− 0.132	− 0.186	− 0.0109	− 0.0396	− 0.112
	(− 1.16)	(− 0.95)	(− 0.68)	(− 0.63)	(− 1.29)	(− 0.22)	(− 0.34)	(− 0.02)	(− 0.07)	(− 0.19)
Legal quality						− 0.00236	− 0.000928	− 0.00196	− 0.00166	− 0.00296
						(− 0.83)	(− 0.39)	(− 0.71)	(− 0.60)	(− 1.16)
Congestion2		0.158***	0.168***	0.171***			0.158***	0.168***	0.171***	
		(4.78)	(4.79)	(5.01)			(4.87)	(4.91)	(5.14)	
Congestion3		0.446***	0.435***	0.439***			0.444***	0.431***	0.436***	
		(8.37)	(8.93)	(9.19)			(8.35)	(9.13)	(9.51)	
Access2		0.0349	0.00176	0.00179			0.0345	0.000571	0.000665	
		(0.99)	(0.06)	(0.06)			(0.96)	(0.02)	(0.02)	
Access3		0.195**	0.143*	0.143*			0.195**	0.142*	0.141*	
		(2.14)	(1.78)	(1.86)			(2.11)	(1.73)	(1.82)	
Access4		− 0.0575	− 0.0903	− 0.0777			− 0.0546	− 0.0852	− 0.0740	
		(− 0.55)	(− 1.10)	(− 0.86)			(− 0.53)	(− 1.02)	(− 0.82)	
Ability1			0.132***	0.142***	0.143***			0.134***	0.143***	0.146***
			(4.53)	(4.96)	(4.12)			(4.52)	(4.95)	(4.19)
Pollution1			0.0187	0.0194	0.0329			0.0224	0.0226	0.0375
			(0.40)	(0.42)	(0.56)			(0.46)	(0.47)	(0.63)
Frequency2				0.0239					0.0235	
				(1.00)					(0.97)	
Frequency3				0.00805					0.00745	
				(0.21)					(0.19)	
Length2				− 0.0105					− 0.0105	
				(− 0.30)					(− 0.29)	
Length3				− 0.0225					− 0.0207	
				(− 0.33)					(− 0.31)	
Wave quality2	0.0707**	0.0572*	0.0403		0.0497	0.0730**	0.0582*	0.0422		0.0529
	(1.99)	(1.91)	(1.25)		(1.42)	(2.07)	(1.93)	(1.29)		(1.50)
Wave quality3	0.114**	0.0821**	0.0369		0.0359	0.114**	0.0821**	0.0364		0.0358
	(2.22)	(2.11)	(0.91)		(0.75)	(2.12)	(2.07)	(0.86)		(0.72)
Tourism1					0.202***					0.199***
					(6.42)					(6.81)
Urban1					0.00410					0.00782
					(0.16)					(0.30)
Observations	750	750	750	750	750	750	750	750	750	750
Prob	0.0113	0	0	0	0	0.0260	0	0	0	0
$Chi^2$	13.00	198.4	168.6	171.5	149.0	12.73	235.5	224.6	248.9	141.1
$R^2$	0.0376	0.136	0.159	0.158	0.114	0.0386	0.136	0.159	0.158	0.116

Standard errors in parentheses: $${*}p<0.10$$, $${**}p<0.05$$, $${***}p<0.01$$

To test formally whether institutions, resource quality, and resource scarcity interact jointly to affect the prevalence of surf localism, I re-estimate the most basic models of Table 2, adding a series of interaction terms in Table 4. Columns (1–3) show interactions for the different institutional measures with wave quality to test for a heterogeneous effect of resource quality, while columns (4–6) show interactions with congestion to account for a heterogeneous effect of resource scarcity. It should be noted that only the interaction with wave quality can be regarded as purely exogenous, though, as congestion might potentially be influenced by the presence of localism.

**Fig. 1:**
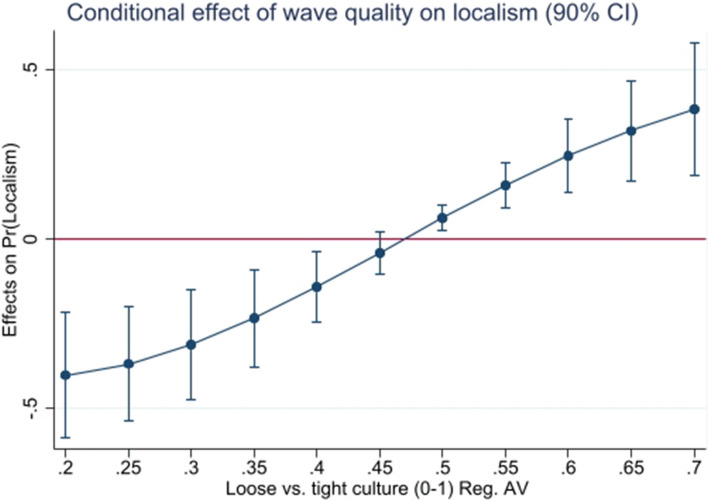
Marginal impact of wave quality by tight-loose cultural norms

Columns (1), (2), and (3) of Table 4 test whether better resource quality interacts with formal and informal institutions jointly to determine the probability of observing localism. Of those three, only the interaction with cultural tighness-looseness in model (2) produces relevant results. Figure 1 is one possible way of interpreting the interactions of both variables in model (2), indicating that a one standard deviation improvement in wave quality increases the occurrence of exclusionary localism in societies with relatively tight social norms. In turn, better wave quality seems to reduce the occurrence of exclusionary localism for societies with relatively loose social norms. So, while cultural tightness-looseness is by itself always an insignificant determinant of localism, the importance that regional cultures place on the importance of respecting norms interacts strongly with resource quality. Those results mirror findings by Gelfand et al. (2021) that tight and loose cultures seem to respond very differently to variations in their external environments.

**Fig. 2:**
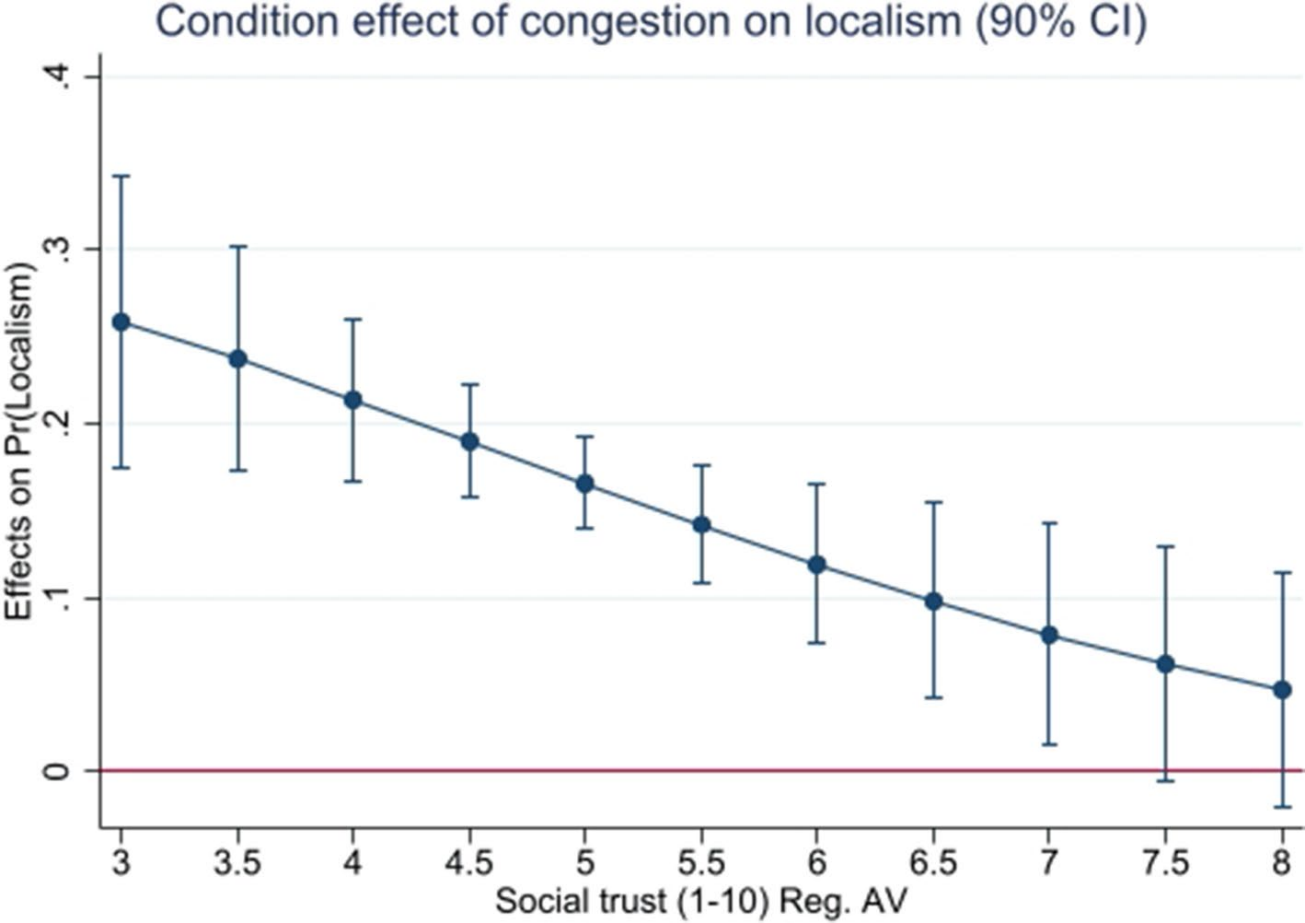
Marginal impact of congestion by social trust

Columns (4), (5), and (6) of Table 4 simply repeat the exercise with resource scarcity as a mediator. Here, only the interaction of congestion with social trust in model (4) produces notable findings. Figure 2 shows graphically the marginal impact of a one standard deviation increase in congestion on the probability of observing localism, depending on the level of social trust. Despite the necessary caution of causally interpreting the graphic, Fig. 2 indicates that an upsurge in congestion would increase the occurrence of exclusionary localism at relatively low levels of social trust. In turn, increasing congestion seems to be irrelevant at comparatively high levels of social trust. To say that in a different way, the exclusionary temptation stemming from increasing resource scarcity seems to be much more severe at lower levels of social trust. Interestingly, this results mirrors findings on the mediating role of trust in other scarcity conflicts, such as those that are present in anti-immigration sentiments (Pellegrini et al. 2021), or physically in the fluctuation of fishing resources for underdeveloped coastal communities (Ramirez-Sanchez and Pinkerton 2009).

**Table 4:** Determinants of localism (Interaction effects)

	(1)	(2)	(3)	(4)	(5)	(6)
Variables	Wave qual.	Wave qual.	Wave qual.	Congestion	Congestion	Congestion
Social trust	0.0685	− 0.192	− 0.227*	− 0.118	− 0.258*	− 0.264*
	(0.25)	(− 1.57)	(− 1.78)	(− 0.43)	(− 1.87)	(− 1.96)
Tight-loose	− 0.471	− 14.09***	− 0.606	− 0.246	1.450	− 0.276
	(− 0.22)	(− 3.06)	(− 0.29)	(− 0.12)	(0.33)	(− 0.13)
Legal quality	− 0.00776	− 0.0117	− 0.00722	− 0.00339	− 0.00278	0.0160
	(− 0.74)	(− 1.07)	(− 0.25)	(− 0.32)	(− 0.27)	(1.00)
Wave quality	0.973	− 3.501***	0.237			
	(1.49)	(− 2.91)	(0.32)			
Soc.trust*Wv.qual.	− 0.160					
	(− 1.24)					
Tight-l.*Wv.qual.		7.454***				
		(3.09)				
Leg.qual*Wv.qual.			− 0.000333			
			(− 0.03)			
Congestion				1.047*	1.205	1.347**
				(1.75)	(1.20)	(2.42)
Soc.trust*Cong.				− 0.0790		
				(− 0.69)		
Tight-l.*Cong.					− 1.112	
					(− 0.56)	
Leg.qual*Cong.						− 0.0109
						(− 1.32)
Observations	750	750	750	750	750	750
Prob	0.00960	0.00103	0.0232	0	0	0
$Chi^2$	15.19	20.44	13.02	115.5	111.1	120.9
$R^2$	0.0408	0.0509	0.0380	0.122	0.122	0.124

Standard errors in parentheses: $${*}p<0.10$$, $${**}p<0.05$$, $${***}p<0.01$$

Finally, all basic regressions of Table 2 are re-run employing a series of robustness checks in the Appendix: First, I check that the institutional macro-variables don’t produce artificial results, perhaps owing to their highly aggregative natures. For that purpose, wave quality is entered as an alternative dependent variable. In that case, neither regional level culture nor legal systems should show up as statically significant because wave quality should be independent of the former variables. Applying logit estimations, Table 5 of the appendix shows that that indeed is the case, greatly enhancing the confidence one can place in the findings corresponding to the macro-variables.

Second, with only 29 regions in the empirical analysis, a risk arises that the findings are driven by a few extreme (regional) observations. Table 6 reports results estimated with bootlegged standard errors, while Table 7 conducts a full jackknife analysis, whereby individual regions are excluded one at a time. In both cases, the empirical findings are remarkably robust and practically identical to those of Table 2. So, despite the relatively small numbers of macro-level observations, the baseline results do not seem to be driven by individual regions that contain extreme clusters of (non-)localized surf spots.

Third, because social trust generally is high in Nordic countries, whereas it is comparatively low in Southern Europe, Table 8 tries to sort out the importance of regional social trust against national-level determinants by entering country fixed effects. Their introduction leaves the impact of social trust on localism always insignificant. In turn, the effect of formal legal quality now turns out to be negative and statistically significant in models (8), (9), and (10). In many ways, governing surf spots in Atlantic Europe through exclusionary localism or the surfer’s code is, therefore, mostly a story of cooperating Nordics versus less-cooperative Southern Europeans, with formal legal institutions perhaps playing a complementary role at the regional level.

## Conclusions

Following studies by Nazer (2004); Kaffine (2009), and Mixon (2014), this paper argues that the sport of surfing is an excellent laboratory for studying open access resources in the context of formally absent property rights and little to no state intervention. Such conflicts can either be solved through exclusion, or voluntary collective rule-following, both of which exist at surf breaks worldwide in the form of localism and reliance on the surfer’s code. While one is a source of pride for most surfers, the other is often regarded as a stain on the sport (Nazer 2004).

This paper relies on surfing localism as a backdrop to ask whether cultural dispositions to cooperate and respect rules determine the adoption of exclusion in common pool resource conflicts, and how such cultural traits interact with resource quality and resource scarcity. In a nutshell, the results show that social trust facilitates compliance with the surfer’s code to manage conflicts over local waves. Although formal state institutions potentially serve a similar function, the evidence presented herein shows them mostly to be irrelevant, despite the fact that well-working legal systems eventually might crowd-in civic behavior. The reported findings further indicate that the tightness-looseness axis of cultural norms interact with resource quality to a substantial degree, while the relevance of social trust increases with more resource scarcity.

In a narrow sense, the findings forwarded in this paper are important for the governance of surf breaks in developing countries, where surf tourism quickly is increasing the returns to the open access resource of surfing waves. That development often sparks local conflicts over high-value surf venues, which often present the sole sources of income for many underdeveloped coastal communities (Ponting and O’Brien 2015; Usher and Gómez 2016). Following recommendations by Nazer (2004), the findings reported above suggest that, where social trust and well-working legals systems largely are absent, assigning property rights over surf breaks to local tribes or other small-scale communities likely will facilitate the surfing venue’s sustainable exploitation, reducing the potential for predatory behavior by outsiders.

In a broad sense, the findings raise much more general questions on how much societies should rely on formal or informal institution to manage resource conflicts that are not in society’s center focus (Coase 1960; Powell and Stringham 2009; Bjørnskov 2011; Skarbek 2014, e.g.). To a large degree, the answer probably depends on how trusting and cooperative local cultures are, as well as on the quality of their formal law enforcement institutions. In that sense, the results also raise important questions for future research on how problems of local governance should best be addressed in areas of weak statehood, where fostering dispositions to cooperate probably can make a substantial contribution to economic development (Börzel and Risse 2016).

## Appendix

See Tables 5, 6, 7 and 8.

**Table 5:** Determinants of wave quality

Variables	(1)	(2)	(3)	(4)	(5)	(6)	(7)	(8)	(9)	(10)
	Culture	Culture	Culture	Culture	Culture	Cult. + Leg.	Cult. + Leg.	Cult. + Leg.	Cult. + Leg.	Cult. + Leg.
Social trust	− 0.159	− 0.120	− 0.0603	0.000928	− 0.140	− 0.461	− 0.441	− 0.299	− 0.253	− 0.423
	(− 0.85)	(− 0.66)	(− 0.34)	(0.01)	(− 0.82)	(− 0.84)	(− 0.79)	(− 0.53)	(− 0.45)	(− 0.79)
Tight-loose	2.622	2.838	2.704	5.137	2.590	− 0.110	− 0.0658	0.559	2.878	0.0676
	(0.38)	(0.41)	(0.37)	(0.75)	(0.36)	(− 0.01)	(− 0.01)	(0.08)	(0.44)	(0.01)
Legal quality						0.0245	0.0261	0.0192	0.0206	0.0227
						(0.53)	(0.55)	(0.40)	(0.42)	(0.50)
Congestion2		0.307*	0.341*	0.256			0.321*	0.351*	0.267	
		(1.72)	(1.87)	(1.47)			(1.77)	(1.88)	(1.52)	
Congestion3		0.268	0.198	0.184			0.299	0.227	0.216	
		(0.81)	(0.63)	(0.63)			(0.85)	(0.66)	(0.68)	
Access2		0.148	− 0.0765	− 0.0696			0.163	− 0.0627	− 0.0527	
		(0.50)	(− 0.27)	(− 0.24)			(0.55)	(− 0.23)	(− 0.19)	
Access3		0.878	0.510	0.387			0.917	0.544	0.426	
		(1.34)	(0.79)	(0.57)			(1.37)	(0.83)	(0.62)	
Access4		1.809*	1.383	1.506			1.729	1.324	1.455	
		(1.65)	(1.29)	(1.53)			(1.58)	(1.24)	(1.47)	
Ability1			0.992***	0.944***	0.997***			0.977***	0.930***	0.983***
			(4.14)	(4.02)	(3.67)			(4.03)	(3.94)	(3.56)
Pollution1			0.134	0.184	0.218			0.108	0.155	0.193
			(0.56)	(0.77)	(0.99)			(0.48)	(0.69)	(0.93)
Frequency2				0.525***					0.533***	
				(3.32)					(3.42)	
Frequency3				0.741***					0.762***	
				(4.74)					(4.92)	
Length2				0.651***					0.647***	
				(3.69)					(3.70)	
Length3				0.747					0.719	
				(1.59)					(1.57)	
Tourism1					0.395*					0.416*
					(1.82)					(1.89)
Urban1					− 0.541**					− 0.556**
					(− 2.44)					(− 2.48)
Observations	750	750	750	750	750	750	750	750	750	750
Prob	0.0113	0	0	0	0	0.0260	0	0	0	0
$Chi^2$	13.00	198.4	168.6	171.5	149.0	12.73	235.5	224.6	248.9	141.1
$R^2$	0.0376	0.136	0.159	0.158	0.114	0.0386	0.136	0.159	0.158	0.116

Standard errors in parentheses: $${*}$$ $$p<0.10$$, $${**}$$ $$p<0.05$$, $${***}$$ $$p<0.01$$

**Table 6:** Determinants of localism (Bootlegged SE)

Variables	(1)	(2)	(3)	(4)	(5)	(6)	(7)	(8)	(9)	(10)
	Culture	Culture	Culture	Culture	Culture	Cult. + Leg.	Cult. + Leg.	Cult. + Leg.	Cult. + Leg.	Cult. + Leg.
Social trust	− 0.332***	− 0.300***	− 0.277***	− 0.266***	− 0.292***	− 0.225	− 0.254	− 0.175**	− 0.180	− 0.145
	(− 4.06)	(− 3.14)	(− 4.66)	(− 3.14)	(− 3.55)	(− 1.52)	(− 1.45)	(− 2.05)	(− 1.24)	(− 0.92)
Tight-loose	− 1.535	− 1.217	− 1.081	− 1.031	− 1.920	− 0.475	− 0.745	− 0.0446	− 0.163	− 0.440
	(− 0.98)	(− 0.58)	(− 0.56)	(− 0.55)	(− 1.27)	(− 0.23)	(− 0.42)	(− 0.02)	(− 0.06)	(− 0.22)
Legal quality						− 0.00848	− 0.00371	− 0.00805	− 0.00681	− 0.0116
						(− 0.75)	(− 0.35)	(− 1.00)	(− 0.67)	(− 1.27)
Congestion2		0.623***	0.678***	0.689***			0.621***	0.674***	0.687***	
		(6.11)	(8.21)	(6.18)			(3.84)	(7.13)	(6.79)	
Congestion3		1.409***	1.425***	1.437***			1.405***	1.415***	1.428***	
		(7.36)	(7.61)	(9.08)			(7.82)	(7.31)	(8.07)	
Access2		0.138	0.00732	0.00741			0.136	0.00237	0.00276	
		(0.94)	(0.06)	(0.05)			(1.01)	(0.02)	(0.02)	
Access3		0.665*	0.513*	0.511*			0.664***	0.507	0.504*	
		(1.83)	(1.78)	(1.83)			(2.82)	(1.63)	(1.84)	
Access4		− 0.265	− 0.446	− 0.372			− 0.250	− 0.415	− 0.351	
		(− 0.43)	(− 0.72)	(− 0.71)			(− 0.35)	(− 0.85)	(− 0.64)	
Ability1			0.508***	0.544***	0.521***			0.515***	0.549***	0.532***
			(3.06)	(3.88)	(4.37)			(3.40)	(4.60)	(4.11)
Pollution1			0.0757	0.0783	0.125			0.0902	0.0908	0.142
			(0.63)	(0.53)	(1.13)			(0.62)	(0.74)	(0.89)
Frequency2				0.0989					0.0972	
				(0.52)					(0.61)	
Frequency3				0.0340					0.0315	
				(0.16)					(0.13)	
Length2				− 0.0433					− 0.0431	
				(− 0.35)					(− 0.34)	
Length3				− 0.0943					− 0.0865	
				(− 0.46)					(− 0.23)	
Wave quality2	0.271*	0.241*	0.170		0.200	0.280**	0.245	0.178		0.214
	(1.86)	(1.78)	(1.22)		(1.56)	(2.38)	(1.42)	(1.25)		(1.37)
Wave quality3	0.413**	0.334**	0.157		0.147	0.415***	0.334	0.155		0.148
	(2.33)	(2.28)	(0.79)		(0.97)	(3.03)	(1.38)	(0.76)		(0.87)
Tourism1					0.720***					0.711***
					(7.93)					(5.00)
Urban1					0.0160					0.0305
					(0.10)					(0.25)
Observations	750	750	750	750	750	750	750	750	750	750
p	0.000258	0	0	0	0	6.20e−07	0	0	0	0
$Chi^2$	21.45	108.8	145.4	483.1	119.0	36.93	161.4	100.2	213.3	84.80
$R^2$	0.0376	0.136	0.159	0.158	0.114	0.0386	0.136	0.159	0.158	0.116

Standard errors in parentheses: $${*}$$ $$p<0.10$$, $${**}$$ $$p<0.05$$, $${***}$$ $$p<0.01$$

**Table 7:** Determinants of localism (Jackknife)

Variables	(1)	(2)	(3)	(4)	(5)	(6)	(7)	(8)	(9)	(10)
	Culture	Culture	Culture	Culture	Culture	Cult. + Leg.	Cult. + Leg.	Cult. + Leg.	Cult. + Leg.	Cult. + Leg.
Social trust	− 0.332**	− 0.300***	− 0.277**	− 0.266**	− 0.292**	− 0.225	− 0.254	− 0.175	− 0.180	− 0.145
	(− 2.48)	(− 3.04)	(− 2.61)	(− 2.50)	(− 2.18)	(− 1.54)	(− 1.31)	(− 0.67)	(− 0.62)	(− 0.71)
Tight-loose	− 1.535	− 1.217	− 1.081	− 1.031	− 1.920	− 0.475	− 0.745	− 0.0446	− 0.163	− 0.440
	(− 1.06)	(− 0.85)	(− 0.57)	(− 0.50)	(− 1.07)	(− 0.19)	(− 0.30)	(− 0.01)	(− 0.05)	(− 0.16)
Legal quality						− 0.00848	− 0.00371	− 0.00805	− 0.00681	− 0.0116
						(− 0.64)	(− 0.24)	(− 0.39)	(− 0.30)	(− 0.76)
Congestion2		0.623***	0.678***	0.689***			0.621***	0.674***	0.687***	
		(4.92)	(4.99)	(5.20)			(4.99)	(5.04)	(5.23)	
Congestion3		1.409***	1.425***	1.437***			1.405***	1.415***	1.428***	
		(8.82)	(8.58)	(8.82)			(9.11)	(9.05)	(9.35)	
Access2		0.138	0.00732	0.00741			0.136	0.00237	0.00276	
		(1.01)	(0.06)	(0.06)			(0.99)	(0.02)	(0.02)	
Access3		0.665***	0.513**	0.511**			0.664***	0.507**	0.504**	
		(3.11)	(2.29)	(2.30)			(3.05)	(2.20)	(2.24)	
Access4		− 0.265	− 0.446	− 0.372			− 0.250	− 0.415	− 0.351	
		(− 0.71)	(− 1.29)	(− 0.91)			(− 0.67)	(− 1.20)	(− 0.85)	
Ability1			0.508***	0.544***	0.521***			0.515***	0.549***	0.532***
			(4.62)	(5.04)	(4.39)			(4.50)	(4.94)	(4.48)
Pollution1			0.0757	0.0783	0.125			0.0902	0.0908	0.142
			(0.47)	(0.47)	(0.49)			(0.53)	(0.52)	(0.55)
Frequency2				0.0989					0.0972	
				(0.90)					(0.87)	
Frequency3				0.0340					0.0315	
				(0.23)					(0.21)	
Length2				− 0.0433					− 0.0431	
				(− 0.28)					(− 0.28)	
Length3				− 0.0943					− 0.0865	
				(− 0.32)					(− 0.29)	
Wave quality2	0.271*	0.241*	0.170		0.200	0.280*	0.245	0.178		0.214
	(1.79)	(1.74)	(1.13)		(1.26)	(1.80)	(1.69)	(1.10)		(1.29)
Wave quality3	0.413**	0.334**	0.157		0.147	0.415**	0.334**	0.155		0.148
	(2.14)	(2.09)	(0.85)		(0.70)	(2.10)	(2.15)	(0.85)		(0.69)
Tourism1					0.720***					0.711***
					(5.83)					(6.15)
Urban1					0.0160					0.0305
					(0.14)					(0.27)
Observations	750	750	750	750	750	750	750	750	750	750
Prob	0.0637	1.84e−09	1.81e−08	1.46e−07	2.69e−09	0.111	4.36e−10	1.85e−09	1.38e−08	6.07e−09
$R^2$	0.0376	0.136	0.159	0.158	0.114	0.0386	0.136	0.159	0.158	0.116

Standard errors in parentheses: $${*}$$ $$p<0.10$$, $${**}$$ $$p<0.05$$, $${***}$$ $$p<0.01$$

**Table 8:** Determinants of localism (Country FE)

Variables	(1)	(2)	(3)	(4)	(5)	(6)	(7)	(8)	(9)	(10)
	Culture	Culture	Culture	Culture	Culture	Cult. + Leg.	Cult. + Leg.	Cult. + Leg.	Cult. + Leg.	Cult. + Leg.
Social trust	0.0877	− 0.143	− 0.277	− 0.338	− 0.143	0.289	0.0891	0.0659	0.0133	0.189
	(0.32)	(− 0.65)	(− 1.09)	(− 1.28)	(− 0.52)	(0.78)	(0.31)	(0.19)	(0.04)	(0.51)
Tight-loose	− 0.119	− 0.633	− 0.871	− 0.134	0.285	1.354	1.028	1.501	2.325	2.544
	(− 0.03)	(− 0.18)	(− 0.21)	(− 0.03)	(0.07)	(0.42)	(0.32)	(0.42)	(0.65)	(0.70)
Legal quality						− 0.0163	− 0.0186	− 0.0269*	− 0.0276**	− 0.0262*
						(− 1.12)	(− 1.42)	(− 1.88)	(− 2.07)	(− 1.77)
Congestion2		0.604***	0.666***	0.689***			0.606***	0.672***	0.692***	
		(4.31)	(4.52)	(4.78)			(4.28)	(4.48)	(4.72)	
Congestion3		1.393***	1.433***	1.464***			1.394***	1.428***	1.459***	
		(8.38)	(8.81)	(9.34)			(8.32)	(8.75)	(9.30)	
Access2		0.133	− 0.0127	− 0.0161			0.128	− 0.0219	− 0.0234	
		(0.97)	(− 0.10)	(− 0.12)			(0.93)	(− 0.16)	(− 0.17)	
Access3		0.654**	0.519**	0.488**			0.668**	0.537**	0.504**	
		(2.48)	(2.02)	(2.04)			(2.56)	(2.11)	(2.15)	
Access4		− 0.325	− 0.478	− 0.429			− 0.304	− 0.447	− 0.388	
		(− 0.66)	(− 1.04)	(− 0.88)			(− 0.63)	(− 1.00)	(− 0.81)	
Ability1			0.543***	0.574***	0.527***			0.560***	0.589***	0.542***
			(4.89)	(5.83)	(4.84)			(5.15)	(6.07)	(5.11)
Pollution1			0.0539	0.0457	0.104			0.0941	0.0907	0.135
			(0.28)	(0.25)	(0.49)			(0.51)	(0.52)	(0.65)
Frequency2				0.142					0.149	
				(1.46)					(1.51)	
Frequency3				0.0550					0.0623	
				(0.34)					(0.40)	
Length2				− 0.0249					− 0.00231	
				(− 0.17)					(− 0.02)	
Length3				0.00165					0.00301	
				(0.01)					(0.01)	
Wave quality2	0.412***	0.347***	0.287**		0.306**	0.411***	0.348***	0.288**		0.306**
	(3.07)	(3.20)	(2.43)		(2.25)	(3.03)	(3.16)	(2.40)		(2.23)
Wave quality3	0.440**	0.359**	0.182		0.173	0.442**	0.362**	0.181		0.173
	(2.22)	(2.29)	(1.00)		(0.82)	(2.15)	(2.22)	(0.94)		(0.78)
Tourism1					0.662***					0.658***
					(5.69)					(5.76)
Urban1					0.00118					0.0221
					(0.01)					(0.21)
Observations	725	725	725	725	725	725	725	725	725	725
$R^2$	0.0650	0.155	0.178	0.175	0.130	0.0665	0.156	0.181	0.178	0.133

Standard errors in parentheses: $${*}$$ $$p<0.10$$, $${**}$$ $$p<0.05$$, $${***}$$ $$p<0.01$$. Note: Observations for Denkmark and Iceland automatically excluded

## Footnotes

1 https://www.pvestates.org/services/police-department/surfer-localism.

2 https://www.surfer.com/features/covid-19-is-spurring-a-new-kind-of-localism/.

3 https://www.surfertoday.com/surfing/how-does-wave-priority-work-in-surfing.

4 Note that the effectiveness of social trust may depend further on common knowledge and acceptance of shared norms.

5 A recent magazine article describes the difficulties of fighting localism through legal systems rather well: https://www.surfer.com/features/battle-for-the-bay/

6 See Powell and Stringham (2009) for an excellent overview of the relevant literature.

7 When double checking was not possible, associated attempts at data manipulation nonetheless often are easy to detect. See, for example, the description of the surf spot Ghost House in Southern Norway.

8 The relevant papers all code observations from the US-based company website www.surfline.com, which provides detailed information on surf spots in North America and other parts of the world.

9 Corresponding roughly to <50 meters, 50–150 meters, >150 meters.

10 They are located in: Belgium, Denmark, France, Iceland, Ireland, Netherlands, Norway, Portugal, Spain, and the United Kingdom.

11 Those results are not reported, but available from the author upon request.

12 The results are not shown, but available from the author upon request.

13 Odds-ratios are not employed, because following Norton and Dowd (2018) average marginal effects generally are preferable for showing the effect of explanatory variables on binary dependent variables, unless the data are from a case-control study.
